# [Corrigendum] Identification of key differentially expressed genes associated with non-small cell lung cancer by bioinformatics analyses

**DOI:** 10.3892/mmr.2026.13976

**Published:** 2026-07-29

**Authors:** Yubo Xiao, Min Feng, Haiying Ran, Xiao Han, Xuegang Li

Mol Med Rep 17: 6379–6386, 2018; DOI: 10.3892/mmr.2018.8726

Subsequently to the publication of the above paper, an interested reader drew to the Editor's attention that the authors may have misstated the datasets used in their study at the start of the Results section. Specifically, at the beginning of the Materials and methods section (in the “*Affymetrix microarray data*” subsection), it is stated that “Series matrix files of the microarray data: GSE21933, GSE33532, GSE44077 and GSE74706 were downloaded from the public GEO database”, whereas in the first sentence of the Results section, in the “*Identification of DEGs*” subsection, it is stated “In order to identify the DEGs, we downloaded 4 public RNA-seq datasets of NSCLC from the GEO database for our analysis: GSE21933, GSE33532, GSE44077 and GSE74706.”

After having considered this comment by the external reader, the Editor of *Molecular Medicine Reports* has judged that the four datasets in question should have also been described as microarray datasets in the Results section of this paper. Although we have been unable to reach the authors in this regard, we are continuing to ask them to confirm that the text in the Results section was indeed written incorrectly. We apologize to the readership of the Journal for any confusion in this matter.

